# Impacts of the COVID-19 Pandemic upon Chinese Positive Traits

**DOI:** 10.3390/ijerph192013490

**Published:** 2022-10-18

**Authors:** Liang Zhao, Yukun Zhao, Yiwen Wu, Xiaojun Ding, Feng Yu, Kaiping Peng

**Affiliations:** 1 School of Information Management, Wuhan University, Wuhan 430072, China; 2 Key Laboratory of Semantic Publishing and Knowledge Service of the National Press and Publication Administration, Wuhan University, Wuhan 430072, China; 3 Positive Psychology Research Center, Tsinghua University, Beijing 100084, China; 4 Department of Psychology, The Chinese University of Hong Kong, Hong Kong 999077, China; 5 Department of Philosophy, School of Humanities and Social Science, Xi’an Jiaotong University, Xi’an 710049, China; 6 Department of Psychology, School of Philosophy, Wuhan University, Wuhan 430072, China; 7 Department of Psychology, School of Social Sciences, Tsinghua University, Beijing 100084, China

**Keywords:** character strengths, COVID-19 pandemic, positive traits, collectivistic culture, disaster

## Abstract

Will Chinese people change in terms of their character strengths when disasters strike? As far as the most recent COVID-19 pandemic is concerned, we provide an explorative answer from the impacts of positive traits included in the Values in Action Classification of Strengths upon Chinese people. We conducted a large-scale online survey from 1 January 2019 to 13 February 2020, with 12,878 respondents nationwide, covering all the administrative regions in China and all age intervals. The changes in the 24 character strengths before and during the pandemic were compared. Results revealed a significant increase in teamwork triggered by the pandemic among Chinese people. Fine-grained differences in demographic variables were also examined. Results showed that the COVID-19 pandemic significantly boosted teamwork for both males and females. Concerning age differences, only younger adults (18–25-year-old) showed a significant increase in teamwork. Besides this, it was also discovered that females always performed a higher teamwork tendency than males, and the elderly higher than the younger, regardless of the pandemic.

## 1. Introduction

Human history is shaped by a series of crises. Disasters bring not only life threats, but also notably dramatic psychological impacts, being them epidemics (e.g., leprosy, schistosomes, etc.) [1], or extremely dramatic outside events (e.g., the 911 terrorist attack) [2]. At the beginning of 2020, COVID-19 rapidly developed into an unprecedented pandemic worldwide, with a globally infected population of over 500 million by 13 June 2022. The pandemic triggers a series of negative impacts upon citizens all over the world [3], including fear of contagion with death threats [4], long- or short-term consequences of psychological distress due to the prolonged social quarantine [5,6], and the death of relatives [7].

Nonetheless, most of our humans are never vulnerable to disaster strikes from the perspective of positive psychology [8]. Positive traits could activate positive experiences and help build the best things and fulfill a good life for healthy people [9,10]. Investigating various cultures around the world, Peterson and Seligman [11] identified 24 character strengths as positive traits that are universal in human nature, and categorized them into six virtues (i.e., wisdom, courage, humanity, justice, temperance, and transcendence). Described and measured in The Values in Action (VIA) Classification of Strengths, the 24 character strengths include creativity, curiosity, openness, love of learning, perspective, bravery, persistence, honesty, zest, intimacy, kindness, social intelligence, teamwork, fairness, leadership, forgiveness, modesty, prudence, self-control, appreciation of beauty, gratitude, hope, humor, and spirituality. They have been proven to be associated with positive psychological dimensions, such as life satisfaction [12,13,14] and stress coping [15]. Character strengths like hope, zest, gratitude, love, and curiosity have shown positive associations with general mental health [16] and negative associations with depression and anxiety [17,18,19,20].

As COVID-19 is concerned, empirical studies have also reported positive relations between character strengths and mental health, indicated by post-traumatic growth during and after the first pandemic wave [21,22,23,24,25]. Character strengths could moderate between COVID-19-related stress and well-being [23,26,27]. Longitudinal studies in Italy [28], Greece [29], and Spain [30] proved the protective role of character strengths to buffer the stress and anxiety from lockdown in quarantine during the pandemic and sustain mental health.

More importantly, distinguished from the traditional conceptualization of personality, character strengths are malleable, by such factors as targeted training, or after traumatic life events [11]. Character strengths always change following major adverse events (e.g., the 911 terrorist attacks) [31], recovery from illness [32], or traumatic experiences [33,34]. Following the September 11 terrorist attacks in 2001, Peterson and Seligman [31] investigated the impact of the attacks upon positive traits among Americans via an online VIA-IS survey and discovered that seven character strengths, including gratitude, hope, kindness, leadership, intimacy, spirituality, and teamwork, showed increases after the attacks. The enhanced character strengths could be seen as an effective coping mechanism [23], making individuals more resilient and helping them to recover from adversity [35].

Compared with the protective effects on mental health, it is quite interesting to look into the dynamic changes in character strengths caused by the pandemic. Unfortunately, few works have been published on this issue. Only one study in a German-speaking area reported the perceived changes rather than real changes in character strengths by comparisons before and post the first wave of the pandemic [22]. On the other hand, as cultures differ along many psychological dimensions [1], people in different cultural backgrounds may react differently to disasters. Taking the individualism-collectivism cultural syndrome as the most significant cultural difference [36], people from individualistic cultures, such as America, are likely to emphasize a high possibility of the personal self; on the contrary, people from collectivistic cultures, such as Asia, Africa, and South America, tend to emphasize the collective self and the connections with the group [37]. Obviously, previous works on character strength changes under disasters [22,31] were both conducted in individualistic cultures. Insights into collectivistic cultures and cross-cultural comparison remain ambiguous.

In this paper, focusing on the COVID-19 pandemic scenario, we wonder whether the pandemic brings any damage or increases the character strengths in China, a typical collectivistic culture. By leveraging large-scale self-report questionnaires online all over China, we carefully compared the changes in character strengths before and during the pandemic. We are aiming to track the public psychological process of a lasting disaster with respect to positive traits, supplement empirical discoveries in a collectivistic culture, and better understand the impacts of the collective pandemic actually existing around every one of us.

## 2. Literature Review

### 2.1. Character Strengths Researches of COVID-19

Literature on the character strengths researches of the COVID-19 pandemic is on the rise [23,38]. Most of the studies focus on the validation of the protective role of character strengths in sustaining mental health and well-being during the lockdown. Taking the 24 strengths as a whole, Liu and Wang [26] discovered character strengths correlated negatively with adolescents’ perceived stress of the COVID-19 pandemic and their depression symptoms. Similarly, the study of [27] (*n* = 269) supported that all the character strengths together could moderate between COVID-19-related stress and well-being especially for people with chronic conditions and disabilities, while the relations were not significant when single strengths or single virtues were concerned. A survey conducted one month after the lockdown began for Italian (*n* = 944) identified strengths such as hope, zest, gratitude, and love, associated strongly with general distress and mental health [38]. Casali et al. [28] converged the 24 character strengths into one factor and the results from a longitudinal study (*n* = 254) showed character strengths had a direct effect on mental health during the second wave of the pandemic by comparing the first and the second wave of the pandemic in Italy. A study of people from Greece (*n* = 354) also proved most of the 24 human strengths were “able to act” to maintain or enhance subjective well-being, especially love, curiosity, persistence, hope, and zest with a strong association with subjective well-being under quarantine [29]. Coincidentally, the longitudinal study [30] conducted for Spain (*n* = 348) grouped the character strengths into five factors (i.e., fortitude, goodness, intellectual, interpersonal, and restraint) and discovered that all the factors positively correlated with life satisfaction and positive affect over a one-month period during the COVID-19 pandemic lockdown.

So far, there are very few studies investigating the detailed changes in the 24 character strengths related to the pandemic. Gander and Wagner [22] measured three kinds of character strengths changes (i.e., self-perceived changes, perceived changes in close others, and the actual differences before and after the first wave of the pandemic) in a German-speaking area by a longitudinal study (*n* = 372) comparing the first and the second pandemic wave. Although most of the character strengths except zest, hope, humor, and self-regulation showed perceived increases in the self and close others, none of the character strengths carried significant changes in actual differences.

### 2.2. Character Strength Measurements in China

The original and most commonly used tool to measure character strengths is the Values in Action (VIA) Character Strengths Survey [39]. It has 240 items, with 10 items for each of the 24 character strengths. Participants rate to what extent each item’s description fits them on a five-point Likert scale. There would be three problems, however, when this survey is applied in China. First, it takes more than 20 min to complete all 240 items. Many participants do not have the patience to complete the survey or even rush to answer, lowering the validity of the final data. Second, the scale is developed based on the life scenarios and descriptions of Western participants. Some items do not relate well to Chinese participants’ lives, so the participants cannot answer them accurately. Third, the Chinese version of the VIA survey is poorly translated, making it difficult for Chinese participants to answer.

Thus, character strength research in China needs more suitable measurement tools. Several Chinese versions of the character strengths questionnaire have been developed. Duan et al. [40] revised the 240-item VIA survey to a 96-item Chinese Virtues Questionnaire-96 (CVQ-96) with most of the items from the original VIA survey developed in the Western context. Wu [41] developed a 76-item scale for 24 character strengths using an open-ended questionnaire and interviews with Chinese college students. Zang et al. [42] developed a 24-item scale, with one item for each character strength, using college students from underdeveloped regions as participants. Some positive psychological questionnaires also involved character strengths, for example, a questionnaire that included 20 character strengths for college students [43], and a questionnaire that included 15 character strengths for elementary school students [44].

There is at least one of the following problems in the prior versions of Chinese character strength questionnaires. First, they are often developed for a specific population, like college students, rather than for the general population [41,42,43,44]. Second, some of them still borrow items from the original VIA survey developed in the Western context, rather than develop items completely from the local context (e.g., [40]). Third, all these questionnaires manage to reduce the number of items, but some of them end up with too few items, which jeopardizes the validity and reliability (e.g., [42]).

### 2.3. The Present Study

To shed light on the changes in character strengths in China during the pandemic, two research questions need addressing: (1) developing a suitable character strengths measurement tool for Chinese culture; (2) discovering and explaining which character strengths would change due to the pandemic, by increasing or decreasing.

According to the well-established findings of the behavioral immune system theory, humans have evolved a range of cognitive, affective, and behavioral mechanisms to prevent infection risk under infectious pathogen threats [45,46,47]. Existing studies found that people tend to perform coping mechanisms such as in-group favoritism and out-group derogation to help avoid exposure to unfamiliar targets that may bring pathogen dangers [48,49,50]. Augmented social identity [49] and increased collectivism tendency [1,51,52,53] were always observed when perceiving threats. Based on the behavioral immune system theory, it has been proven that under the COVID-19 threat people tended to seek more connections with in-group members and showed augmented communion motives [54].

Inspired by the above self-/group-oriented perspectives, we adapted Haidt’s binding/individualizing categorizations [55] on character strengths. Considering whether or not each character strength fosters interpersonal connections, we categorized the 24 character strengths into two kinds, the binding traits, and the individualizing traits. The former represents the positive traits that reflect how we get along and connect with others, consisting of intimacy, kindness, social intelligence, teamwork, leadership, forgiveness, and gratitude. The latter represents the traits that put more emphasis on personal development and consists of the other 15 character strengths. Therefore, we formulate our hypothesis for the second research question as follows.

**Hypothesis:**  *Some of the binding traits of character strengths that reflect strong interpersonal connections (i.e., intimacy, kindness, social intelligence, teamwork, leadership, forgiveness, and gratitude) will increase during the COVID-19 pandemic.*

## 3. Study 1: Development of Chinese Character Strengths Questionnaire

To better fit into the Chinese context and investigate the impacts on character strengths of COVID-19, the primary job is to develop a Chinese Character Strengths Questionnaire that is based on the theoretical framework of character strengths [11] but aligns with the Chinese context. The number of items should be neither too less nor too many so that the questionnaire keeps good psychometric properties and is meanwhile convenient enough to use without too much burden for users.

### 3.1. Measurement of Character Strengths under Chinese Culture

Based on the theoretical framework of [11] and referring to the existing character strengths and virtues questionnaires in China (e.g., the Chinese version of the VIA survey [39]; the Chinese version of the VIA Youth Survey [43,44,56,57]), we developed Chinese Character Strengths Questionnaire (CCSQ) to measure the 24 character strengths of Chinese people. We conducted interviews with master and doctoral students in the Department of Psychology at Tsinghua University. From their description of the 24 character strengths under Chinese culture, 72 items suitable for both adults and adolescents under the Chinese context were chosen to formulate the Chinese Character Strengths Questionnaire (CCSQ). That is, the CCSQ contains 72 items, with each character strength corresponding to three items. Each item describes the personality matched to a certain character strength, such as “I often express my gratitude to others (grateful)”, “When others do me harm, I will not remember it for a long time (forgiveness and mercy)”, “I love learning new knowledge (love of learning)”. Respondents rate to what extent they agree or disagree with the description of each item on a seven-point Likert scale, from 1, very much unlike me, to 7, very much like me. Scale scores for the character strengths are calculated by averaging the relevant items.

### 3.2. Validations of Chinese Character Strengths Questionnaire

#### 3.2.1. Participants

A total of 5801 participants in China were recruited and they completed the CCSQ via smartphone APP and WeChat web pages through mobile internet from April 2016 to February 2018. The participants with missing values on their answers were deleted from the dataset. In total, there were 3734 females (64.4%) and 2067 males (35.6%). The participants aged between 15 and 68, with an average of 31.63±11.87, from all of the 34 administrative regions in China.

#### 3.2.2. Data Analysis

We first conducted a confirmatory factor analysis (CFA) to confirm the structure of the 24 character strengths. Then we conducted an exploratory factor analysis (EFA) to examine the factorial structure (i.e., the virtues) of the 24 character strengths. Finally, we analyzed the internal consistency of the scale over strengths as well as virtues. Both EFA and internal consistency were analyzed in SPSS 21.0, and CFA was accomplished with AMOS 21.0.

### 3.3. Results

#### 3.3.1. Confirmatory Factor Analysis (CFA) of CCSQ

Based on Peterson and Seligman’s theoretical model [11], 24 character strengths were modeled as 24 correlated latent variables to conduct CFA. We adopted various goodness of fit indices, including $\chi^{2}$ ratio ($\chi^{2}/df$), the comparative fit index (CFI), the goodness of fit index (GFI), the adjust goodness of fit index (AGFI), the Tucker–Lewis index (TLI), root of mean square residual (RMR), and root-mean-square error of approximation (RMSEA), etc. An acceptive model fit holds when $\chi^{2}/df<3$, CFI, GFI,AGFI, TLI≥0.90, and RMR<0.1, RMSEA<0.05 [58]. As shown in Table 1, χ2/df < 3, *REMSEA* < 0.05, *RMR* < 0.1, and the rest of the indicators were all above 0.95, showing a good fit for the model.

The factor loadings of the 72 items were between 0.459 and 0.651 (all *p* < 0.001), indicating that all the latent variables (the 24 character strengths) are meaningful to the measured variables. The normalized variances of 24 latent variables and 72 residuals were all positive (all *p* < 0.001), indicating that the model has no definition errors.

#### 3.3.2. Explanatory Factor Analysis (EFA) of CCSQ

Preliminarily, we checked that the Kaiser–Meyer–Olkin measure of sampling adequacy was higher than 0.50 (KMO = 0.937), and Bartlett’s Test of Sphericity was statistically significant (*p* < 0.001). By Kaiser’s criterion of eigenvalues higher than 1, principal component analysis results indicated that five factors could be extracted, explaining 62.51% of the total variance. As the factors were moderately intercorrelated, the EFA results with an oblique rotation were shown in Table 2.

The first factor explained 38.554% of the total variance and included seven interpersonal-related strengths, such as zest, love, kindness, appreciation of beauty, gratitude, and hope. The second factor explained 7.896% and covered six intellect and transcendence strengths, for example, creativity, curiosity, love of learning, bravery, honesty, and spirituality. The third factor explained 6.228%, including four strengths of temperance, like openness, persistence, prudence, and self-control. The fourth factor explained 5.506% and was loaded by three strengths of justice, such as fairness, forgiveness, and modesty. The last factor explained 4.324%, covering four emotional intellect strengths, perspective, social intelligence, leadership, and humor. Such factor (virtue) structures share similarities with Peterson and Seligman’s initial model [11] and other validations (e.g., [59]), and meanwhile reveal small differences in Chinese contexts (i.e., the number of virtues and component strengths of each virtue differ), which suggests the necessity of adapting the scale to fit the Chinese context.

#### 3.3.3. Internal Consistency of CCSQ

Internal consistency of the scale was tested to be α=0.961. Shown in Table 3, more specifically, for the 18 strengths, the results ranged from α=0.710 to α=0.872, indicating satisfactory reliability. The other six strengths, creativity (α=0.640), openness (α=0.685), honesty (α=0.629), teamwork (α=0.642), appreciation of beauty (α=0.666), spirituality (α=0.658) showed marginal reliability. In general, the results showed adequate reliability for almost all strengths. In addition, for the five virtues (factors) from the EFA result, the internal consistency achieved 0.910, 0.876, 0.873, 0.811, 0.883, respectively.

### 3.4. Discussion

This study developed and validated the Chinese Character Strengths Questionnaire. The questionnaire meets the four goals of design. First, as with the adaptations under other cultures (e.g., [59] for Greece), it fits in a similar theoretical framework as Peterson and Seligman with the 24 strengths [11] but also reflects several cultural differences when structured to the virtue level [59]. Second, it yields satisfactory psychometric properties and factorial structure with promising Cronbach’s α coefficient, and stability coefficient results. Third, it is totally developed under the Chinese cultural context. Fourth, the scale is relatively short and easy to use. Furthermore, it was tested to be effective to use and spread via mobile internet, which would be more and more widespread scenarios for psychological measurements in the future. The CCSQ could serve as a good instrument for character strength research and applications in Chinese culture.

## 4. Study 2: Analyzing the Impacts on Character Strengths of COVID-19 with CCSQ

### 4.1. Participants and Descriptive Statistics

From 1 January 2019 to 13 February 2020, 12,878 Chinese individuals completed the CCSQ online, either via smartphone APP or by WeChat webpages, of which 26.82% were males and 73.18% were females. The participants were from all over China, covering a total of 34 administrative regions nationwide. For privacy protection, we did not ask them about their exact ages, but offered coarse-grained age ranges for them to choose from (i.e., <18, 18–25, 26–30, 31–40, 41–50, 51–60, 61–70, 71–80, and >80). The participants covered all age ranges, with a majority from the younger generation (≤40, occupying 82.54%). Their jobs were also collected as a kind of demographic information. The Cronbach’s alpha of CCSQ was 0.958, indicating the feasibility of the scale.

According to the Chinese government’s official announcement of the outbreak of COVID-19 in China on 20 January 2020, we set 1 January 2019 to 19 January 2019 as Pre-Disaster and 20 January 2020 to 13 February 2020 as During-Disaster. In total, 9239 valid reports (71.74%) were collected in the Pre-Disaster period while 3639 (28.26%) in During-Disaster. Note that in our study, the During-Disaster period only covered from 20 January 2020 to 13 February 2020. However, according to the statistics of infections by the China Center for Disease Control (CDC), this period is within the severest period as the infections dramatically increased every day [54]. Thus, the data and the corresponding results of During-Disaster are typical and reliable enough to reflect the psychological impacts of the pandemic.

To track the detailed changes of the 24 character strengths after the outbreak, we further split the 25-day During-Disaster period into two consecutive sub-intervals divided by the calendar month, the first 12 days from 20 January 2020 to 31 January 2020 denoted as Period-1, and the rest 13 days from 1 February 2020 to 13 February 2020 denoted as Period-2.

### 4.2. Statistical Analysis

The COVID-19 impact on positive traits was analyzed with both SPSS 21.0 and Python 3.6 library *stats*. To check whether the disaster left general impacts on the positive traits, first, we conducted a one-way multivariate analysis of the variance of Pre-Disaster and During-Disaster as the two-level between-subjects factor and the 24 scale scores as dependent variables. Then we dove into the more specific changes in each character’s strength, respectively. In the previous work of Peterson and Seligman [31], to obtain the robust changes of character strengths triggered by the disaster (i.e., the 911 attacks), they comprehensively considered the character strengths changing within two consecutive sub-intervals after the attacks to make the conclusion. Similarly, in our study, for each character strength, we compared the values of Period-1 and Period-2 with that of Pre-disaster by independent-sample *t*-tests, respectively. The effect size was measured by the absolute value of Cohen’s d. The character strengths, which changed significantly and robustly in both sub-intervals during the disaster (i.e., Period-1 and Period-2) with the effect size being bigger than 0.15 (i.e., at least with a medium effect), would be concluded as impacted by the pandemic. Finally, the differences in gender and age over the impacted character strengths in Pre-/During-Disaster were examined by *t*-tests to comprehensively understand the disaster impact upon Chinese positive traits as well.

### 4.3. Sensitivity Analysis of Gender Skew

Specifically, as the female participants occupied the majority of the subjects in our whole dataset, we conducted a sensitivity analysis of the gender distribution to explore the potential impact of the gender skew and validate the robustness of the results. We randomly sampled the female subjects to generate a new subset where the number of females was strictly controlled equally to those of the males in Pre-Disaster/Period-1/Period-2, respectively. Thus, in the newly generated subset, the number of males and females kept equal not only in total but also in each disaster period. Without loss of generality, we denoted the sampled subsets containing the equal size of males and females as gender-controlled sets. For example, as the sizes of the male group in Pre-Disaster/Period-1/Period-2 were 2635/130/754, respectively, we randomly sampled the same number of females in corresponding periods and finally obtained a gender-controlled set of 7038 records (=(2635 + 130 + 754) × 2). Upon each gender-controlled set, we then checked the pandemic impacts via the changes of the 24 character strengths in the same manner as what we did to the whole dataset. To guarantee statistical significance, the sampling and analysis procedure was repeated 20 times. The widely used measurement, confidence [60] was calculated to show, that given the gender-controlled sets, how many times out of 20 would the conclusions of positive trait change be detected as the same as that of the whole dataset. The confidence values were from 0 to 100%. Obviously, a confidence value approaching 100% suggests little impact of the gender skew, otherwise, the gender skew puts a strong impact on the results with the confidence close to 0.

### 4.4. Results

#### 4.4.1. Data Validation

**Figure 1:**
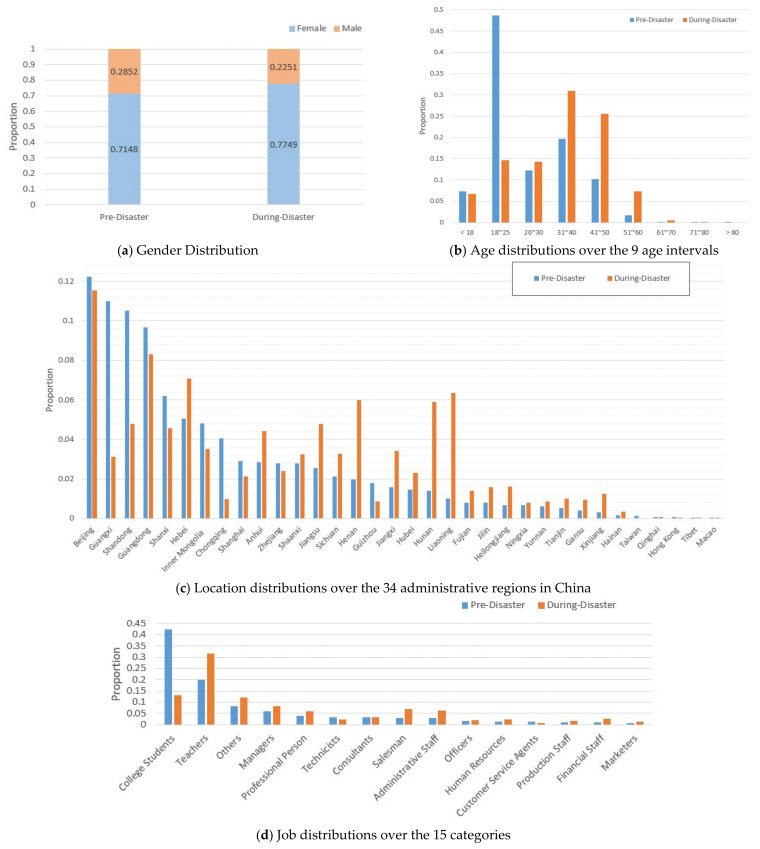
The distributions of demographic variables of the dataset. (**a**) The gender distribution, represented by the proportions of female and male subjects in Pre-/During-Disaster, respectively. (**b**) The age distribution, represented by the proportions of the 9 age intervals in each period. (**c**) The distributions of the subjects’ locations, covering the 34 administrative regions in China. (**d**) The distributions of the subjects’ jobs, covering the 15 major categories. Note that for each period (Pre-/During-Disaster), the distributed proportions of a given demographic variable added up to 1.

Figure 1 compares the distributions of demographic variables (i.e., gender, age, location, job). Obviously, due to the large-scale sampling, such demographic variables were comparable in the two periods, sharing approximately the same distribution tendency. For example, before and during the pandemic, the females both made up around 75% (Figure 1a), and the recruited participants shared the same 14 main categories of professions (Figure 1d). We need to note two exceptions on age and location background. As age is concerned, there were no records in During-Disaster with age > 80. However, since there were only 0.01% of data with age > 80 in the Pre-Disaster period (see Figure 1b), whose size was too small and would not be included for statistical analysis, the age difference between the two periods could be ignored. Similarly, for the location information, there were no records in the During-Disaster period of Taiwan, for which the difference could also be ignored for similar reasons (see Figure 1c). Thus, in general, demographic variables were controlled and comparable in our data, making the following statistical analysis reliable and robust.

#### 4.4.2. General Results

Taking Pre-Disaster and During-Disaster as the two-level between-subjects factor and the 24 scale scores as dependent variables, the result of the one-way analysis of variance was significant, with F(1, 12876) = 569.86, *p* < 0.001, $\eta_{p}^{2}$ = 0.516, indicating actual changes in character strengths triggered by the pandemic.

Table 4 illustrates the fine-grained differences for each character strength in Period-1/Period-2 against Pre-Disaster periods. As mentioned in Section 4.2, if a trait gets significant changes in both Period-1 and 2 with Cohen’s d bigger than 0.15, only then do we consider that the pandemic has an impact on it. It is easy to find the trait teamwork significantly and robustly increased in both sub-intervals during the disaster compared to that of Pre-Disaster, with *t* = 2.0284, *p* = 0.043, *Cohen’s d* = 0.3903 in Period-1, and *t* = 8.7192, *p* < 0.001, *Cohen’s d* = 0.1717 in Period-2. Although social intelligence appeared to get significant decreases in both Period-1 and Period-2, the effect size in Period-2 was smaller than the given threshold of 0.15, which did not meet our criterion. Sensitivity analysis of the gender variable showed that gender skew has no impact on the above conclusions, validating the robustness of the results. The results of the 20 gender-controlled sets showed the same conclusion that only the binding trait, teamwork, increased during the disaster with the confidence of 100%, coinciding with the above results upon the whole dataset (please refer to Table A1a–e in Appendix A for details, where we randomly show the results of five gender-controlled sets). When social intelligence was concerned, its effect size during Period-2 still never reached 0.10 (<0.15). Even without considering the effect size, there were no stable changes in social intelligence in both Period-1/2. For example, as shown in Table A1b,e, only during Period-1 did social intelligence appear to significantly decrease.

Thus, we draw the conclusion that the disaster leads to robust increases in one binding trait, teamwork, with an average effect size of 0.281, and our hypothesis is then validated.

#### 4.4.3. Impacts of Demographic Variables

**Figure 2:**
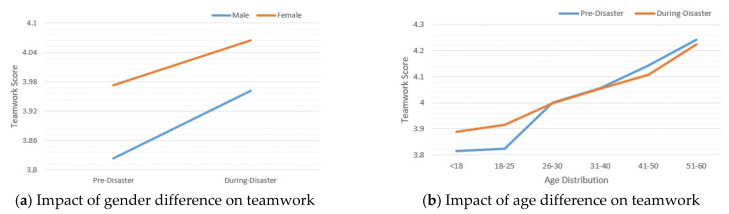
The impacts of demographic variables (gender and age) on teamwork. (**a**) The impact of gender difference on teamwork. (**b**) The impact of age difference on teamwork. The *y*-axis represents the average score of teamwork for each group, valued in [28,60].

Figure 2a compares the gender difference in teamwork (in the whole dataset). Results showed that gender was statistically related to the changes in teamwork scores. Compared with that of Pre-Disaster, the disaster significantly boosted the level of teamwork for both males (*M_During_* = 3.9613, *SD_During_* = 0.6430) and females (*M_During_* = 4.0649, *SD_During_* = 0.6319) with (*t* = 5.3167, *p* < 0.001, *Cohen’s d* = 0.2101), and (*t* = 6.4578, *p* < 0.001, *Cohen’s d* = 0.1451), respectively. It was discovered that females always showed a higher teamwork tendency than males. Even without COVID-19 pandemic impact, just taking Pre-Disaster as a general time period, females (*M* = 3.9733, *SD* = 0.6305) inherently had a significantly higher teamwork score than males (*M* = 3.8230, *SD* = 0.6730) with *t* = 9.8609, *p* < 0.001, *Cohen’s d* = 0.2305.

Figure 2b illustrates the change in teamwork scores for different age groups (i.e., <18, 18~25, 26~30, 31~40, 41~50, 51~60). Specifically, we ignored the age groups of 61~70, 71~80, and >80 whose sample sizes were too small (≤30 each). Compared with that of the Pre-Disaster period, a significant increase of teamwork in During-Disaster could only be observed for those 18~25 years in age (*M_Pre_* = 3.8237, *SD_Pre_* = 0.6247, *M_During_* = 3.9160, *SD_During_* = 0.6170) with *t* = 3.2901, *p* = 0.001, *Cohen’s d* = 0.1494. Similar to the gender difference, age was also statistically related to teamwork changes, where the teamwork score increased with the growth of age.

### 4.5. Discussion

The results from the large-scale online survey revealed that the character strength of teamwork showed an increase during the COVID-19 pandemic in China. The conclusion remains robust regardless of gender skew, suggesting that teamwork appears to be quite a common trait in coping with disasters for both males and females. Meanwhile, as teamwork could be considered a binding trait, reflecting interpersonal connections, our observations are in line with the behavioral immune system theory as we assumed that people will turn to connect more with in-group members when perceiving pathogen threats [45,46,47]. We need to argue that although we also observed social intelligence decreased during the pandemic in some cases, it is not reliable enough to be conclusive due to its inadequate effect size (<0.10 all the way in Period-2) and uncertainty (i.e., non-significant changes in 40% cases). The reason probably lies in the fact that the participants stayed in quarantine and suffered social isolation during both Period-1 and 2 [5,6], which greatly brought a negative influence on the self-assessment of social intelligence. In order to distinguish the pandemic impacts and such real-world noise impacts as much as possible, this is also why we set the pandemic impact holds when a strength continuously changes in both during-disaster periods with considerable effect size.

Furthermore, by investigating the fine-grained changes of teamwork under different gender and ages, we comprehensively understood the pandemic impact. It is interesting that gender was statistically related to teamwork, where females generally performed with a higher teamwork level than males both before and during the pandemic. It may be due to the fact that different from males who adopt a fight-or-flight strategy, females are more likely to adopt the tend-and-befriend biobehavioral mode [57], which corresponds with a higher teamwork level. As pandemics are collective experiences impacting individuals, families, and communities [61], both females and males show a significant increase in teamwork during the pandemic. This could also explain why gender skew had no impact on the conclusions as both genders were affected by the pandemic.

The present results also suggest that age is positively related to teamwork. Compared with younger adults, the elderly population performed at a higher teamwork level either before or during the pandemic. This finding comes in line with the literature, according to which, the interpersonal virtue of people aged 45 plus was found to reach higher levels compared to the younger [59]. Researchers even argued that, except for the intellect virtue, all the other virtues get higher with age [29]. Moreover, our results extend those conclusions and provide further insights into the COVID-19 scenario. When comparing the changes before and during the pandemic, we found the younger groups saw a significant fluctuation in teamwork level compared to the elderly. That is, the pandemic only triggered significant changes (increase) in teamwork in those aged 18~25. This may be due to the fact younger people experience more threats and stress during COVID-19 than the elderly [23,62] so, they perform the most significant coping reactions by arousing character strengths.

## 5. General Discussion

In this paper, with a large and demographically online survey in China, we leveraged a quasi-experimental approach to investigate the character strengths changes before and during the pandemic. Important demographic variables like gender and age were also carefully studied in our analysis. Compared with the original VIA Character Strength Survey [39] and existing Chinese version measurement tools [40,41,42,43,44], we novelly developed the CCSQ scale that is more applicable to the Chinese context and the vast majority of Chinese population, providing effective measurement of Chinese character strengths. Moreover, our data was widely sampled all over China and reached a much larger scale (>10,000), with 9239 before the pandemic and 3639 during the pandemic, than the existing studies (<1000, e.g., [22,26,27,28,30,38]). Specifically, the samples during the pandemic were collected from 2020/1/20 to 2020/2/13. Although the period is not long enough, it covers the most severe period of the pandemic in China [54] and is thus able to reflect the typical psychological changes brought by the pandemic.

The COVID-19 pandemic is a kind of collective experiences impacting individuals and all the citizens. As we know, important experiences and life events are usually tied to slow changes in personality changes across the lifespan [63]. However, our results that the immediate changes in character strengths during the pandemic happened so fast does not conflict with the above common sense. On one hand, the conceptualization of character strengths differs from the traditional personalities, that they are malleable by targeted training or after traumatic life events [11] and easier to change. On the other hand, as an emerging body of work has demonstrated the protective role of character strengths as a coping mechanism to buffer between the negative impacts of the pandemic and mental health [23,26,27,29,35], the observed changes in character strengths in our study might be temporary and reflect more of a situational reaction to hard times. In addition, despite suffering the negative impacts like health threats, stress and depress [3], exposure to traumatic events like the pandemic could also associate with positive outcomes [61]. Our results just illustrate one of the positive parts, as the findings in our work revealed character strength of teamwork performed an increase during the pandemic, which empirically supports the viewpoint that character growth might occur following the experience of trauma [33] in COVID-19 scenario. Based on such findings, psychologists and educators could in turn promote better and more targeting trainings or coping mechanisms by teamwork (e.g., encouraging voluntary services and mutual help during COVID-19 [22]), activate character strengths to foster a positive reinterpretation and buffer the negative impacts of the pandemic [35].

Our work extends the previous findings in terms of granularity and provides more detailed insights. Different from most of the existing COVID-19-related studies that consider the 24 character strengths as a whole [26,27,28] or packaging them into virtues [30,38], we dived into the fine-grained character strengths and shed light upon the robust changes of character strengths during COVID-19. When the comparisons before and during the pandemic are concerned, although Gander and Wagner [22] argued no actual difference was found in German subjects, we discovered significant changes in teamwork by objective CCSQ measurement in Chinese subjects. To the best of our knowledge, this is the first work reporting the actual differences in character strengths before and during the pandemic, greatly bridging the gap between the objectively measured character growth and the COVID-19 pandemic.

Our study additionally contributes to previous literature on positive traits and disaster impacts in the following two aspects as well. First, to supplement the previous work of Peterson and Seligman [31] that positive traits including teamwork increased after the disaster of the 911 attacks in the individualistic culture of America, we took the COVID-19 pandemic as the disaster scenario and contributed the first empirical study with similar conclusions in the collectivistic culture of China. The coinciding conclusions in different cultures may insightfully inspire interesting theoretical explorations of whether teamwork is one of the cross-culturally common character strengths in human nature that function to cope with various kinds of collective disasters. Second, as mentioned in Section 2.3, the behavioral immune system theory [45,46,47] inherently explains psychological phenomena related to infectious diseases and pandemics. Our results revealed an increased level of teamwork during the pandemic and verified the hypothesis of behavioral immune system theory. Thus, under the COVID-19 scenario, our results provide a potential explanation mechanism for pandemic-related character growth from the perspective of BIS theory.

Of course, this study has several limitations we would like to discuss. Firstly, we need to point out that the effect size of the change of teamwork before and during the pandemic in our study was small by conventional standards (<0.3). This may be due to the fact that our study was cross-sectional, not longitudinal designed. Because of the unpredictability of disasters, it is difficult to track large-scale longitudinal studies before and during the disaster [64]. We tried to enlarge the sampling scale which achieved two orders of magnitude more than any previous study (e.g., [26,27,28,30,38]) to reach the wider population in China, reduce the sampling bias and thus approximate statistically longitudinal analysis. As mentioned above, our online survey widely covered populations of similar demographics in both Pre-/During-Disaster periods, making the results convincing. Unfortunately, we could not compare the effect size with the previous work of [31] which was also cross-sectionally designed, as they did not report the effect size. Perhaps in a future study, we could further refine the data in proportion to the age and region distribution of Chinese population to control the sampling bias in age and region. Secondly, our data in this study was collected until 13 February 2020, the early stage after the outbreak of COVID-19. Continuous data collection in a longer lasting period during the pandemic could provide more insights into the dynamic changes of the positive traits in the whole process of the pandemic wave. Thirdly, the data size could be improved over the location dimension. As China is quite a large country, it would be interesting to investigate region differences. Although our data covered all the administrative regions in China, the data size was not big enough to afford the fine-grained region comparisons.

## 6. Conclusions

Focusing on the most recent COVID-19 pandemic, we conducted a large-scale online survey in China nationwide, aiming to shed light on whether such disasters will bring changes in positive traits of the Chinese population. The statistical conclusion is that the character strength of teamwork robustly increased during the COVID-19 pandemic. The results also revealed that affected by the pandemic, both males and females performed at an increased level of teamwork. Concerning age differences, only younger adults (18–25-year-olds) showed a significant increase in teamwork. Besides this, females always performed at higher teamwork levels than males, and the elderly higher than the younger, no matter if it was before or during the pandemic.

## Figures and Tables

**Table 1:** CFA results (*n* = 5801).

Indicators	*χ2*	*df*	*χ2/df*	*GFI*	*AGFI*	*NFI*	*CFI*	*IFI*	*RMR*	*RMSEA*
**Values**	4978.6	2208	2.25	0.976	0.972	0.957	0.976	0.976	0.040	0.015

**Table 2:** Oblimin rotated solution of Principal Component Analysis for CCSQ (*n* = 5801).

Strengths	Factor 1	Factor 2	Factor 3	Factor 4	Factor 5
**Zest**	**0.537**				
**Love**	**0.755**				
**Kindness**	**0.734**				
**Teamwork**	**0.455**				
**Appreciation of beauty**	**0.633**				
**Gratitude**	**0.746**				
**Hope**	**0.778**				
**Creativity**		**0.842**			
**Curiosity**		**0.723**			
**Love of learning**		**0.561**			
**Bravery**		**0.759**			
**Honesty**		**0.630**		0.533	
**Spirituality**		**0.400**			
**Openness**			**0.554**		
**Persistence**			**0.556**		
**Prudence**			**1.017**		
**Self-control**			**0.783**		
**Fairness**				**0.628**	
**Forgiveness**				**0.669**	
**Modesty**				**0.667**	
**Perspective**					**0.524**
**Social intelligence**					**0.682**
**Leadership**					**0.406**
**Humor**					**0.821**
**Eigenvalues**	9.253	1.895	1.495	1.322	1.038
**Variance explained (%)**	38.554	7.896	6.228	5.506	4.324

*Note.* Bold indicates the highest factor loadings for each strength. Factor loadings smaller than 0.40 are eliminated.

**Table 3:** Internal Consistency Coefficients for the Character Strengths (*n* = 5801).

Cronbach’s α Coefficient							
Creativity	0.640	Persistence	0.784	Teamwork	0.642	Self-control	0.712
Curiosity	0.796	Honesty	0.629	Fairness	0.748	Appreciation of Beauty	0.666
Openness	0.685	Zest	0.795	Leadership	0.859	Gratitude	0.730
Love of learning	0.786	Intimacy	0.720	Forgiveness	0.734	Hope	0.848
Perspective	0.816	Kindness	0.863	Modesty	0.713	Humor	0.872
Bravery	0.710	Social intelligence	0.773	Prudence	0.782	Spirituality	0.658

**Table 4:** Mean scores of the 24 character strengths compared before and during the disaster.

Traits	Mean Pre-Disaster (*n* = 9239)	Mean Period-1 (*n* = 280)	d Period-1	Mean Period-2 (*n* = 3359)	d Period-2
Creativity	2.7437 (0.7523)	2.7262 (0.7593)	−0.0233	2.7166 (0.7385)	−0.0365
Curiosity	3.6337 (0.8366)	3.6071 (0.8704)	−0.0312	3.6201 (0.8639)	0.0160
Openness	3.5643 (0.7345)	3.4762 (0.8034)	−0.1146	3.5924 (0.7458)	0.0378
Love of learning	3.8829 (0.7696)	3.8333 (0.7172)	−0.0667	3.9671 (0.7694) ***	0.1094
Perspective	3.5367 (0.7869)	3.5833 (0.8825)	0.0556	3.5539 (0.8065)	−0.0215
Bravery	3.3723 (0.7647)	3.2857 (0.5571)	−0.1295	3.4520 (0.7836) ***	0.1030
Persistence	3.5755 (0.7868)	3.7024 (0.7105)	0.1692	3.6335 (0.8034) ***	−0.0728
Honesty	3.5396 (0.7461)	3.5833 (0.7941)	0.0566	3.6684 (0.7322) ***	0.1741
Zest	3.6312 (0.8725)	3.6905 (0.7477)	0.0729	3.7138 (0.8760) ***	0.0944
Intimacy	4.0262 (0.8003)	3.9643 (0.8081)	−0.0771	3.9433 (0.7914) ***	−0.1043
Kindness	4.2729 (0.6883)	4.3571 (0.4963)	0.1405	4.4030 (0.6371) ***	0.1963
Social intelligence	3.5112 (0.7848)	3.0714(0.7664) **	−0.5671	3.4410 (0.7904) ***	−0.0893
**Teamwork**	**3.9304 (0.6465)**	4.1785 (0.6251) *	**0.3903**	4.0405 (0.6358) ***	**0.1717**
Fairness	3.6925 (0.8205)	3.7500 (0.7122)	0.0748	3.7527 (0.8157) ***	0.0736
Leadership	3.4344 (0.8215)	3.3690 (0.7822)	−0.0817	3.4646 (0.8149)	0.0368
Forgiveness	3.3365 (0.9054)	3.1786 (0.8818)	−0.1768	3.4005 (0.918) ***	0.0701
Modesty	3.8388 (0.8051)	3.7500 (0.9585)	−0.1004	3.8563 (0.8147)	0.0215
Prudence	3.5635 (0.7926)	3.6548 (0.7453)	0.1187	3.5429 (0.8070)	−0.0258
Self-control	3.4072 (0.7982)	3.4167 (0.7731)	0.0120	3.4970 (0.7839)	0.1134
Appreciation of beauty	3.9369 (0.7834)	3.8095 (0.8034)	−0.1606	3.9866 (0.7477)	0.0649
Gratitude	4.0670 (0.7257)	4.2976 (0.5690)	0.3537	4.0910 (0.7262)	0.0331
Hope	4.2035 (0.7784)	4.3690 (0.6109)	0.2366	4.2965 (0.7477)	0.1219
Humor	3.4053 (1.0070)	3.3810 (0.9968)	−0.0244	3.1572 (1.0192) ***	−0.2450
Spirituality	3.9320 (0.7061)	3.9762 (0.7366)	0.0613	3.9747 (0.7169) ***	0.0600

*Note*. Standard deviations are in parentheses. * means *p* < 0.05, ** means *p* < 0.01, *** means *p* < 0.001.

## Data Availability

Data will be provided by the authors on request.

## Appendix A. Sensitivity Analysis of Gender Skew

As female participants occupied the majority of the subjects in our whole dataset. To investigate the potential impact of the gender skew, we further randomly sampled the female subjects to generate a new subset where the number of female participants was strictly controlled equally to that of the males in Pre-Disaster/Period-1/Period-2, respectively. Without loss of generality, we denoted the sampled subsets containing the equal size of males and females as gender-controlled sets. For each gender-controlled set, we then checked the pandemic impacts via the changes of the 24 character strengths in the same manner as what we did to the whole dataset. Table A1a–e showed the mean scores of the 24 character strengths compared before and during the disaster for the five gender-controlled sets, which were randomly sampled.

**Table A1:** Mean scores of the 24 character strengths compared before and during the disaster upon the 5 gender-controlled sets.

(a) Gender-Controlled Set 1					
Traits	Mean Pre-Disaster (*n* = 5270)	Mean Period-1 (*n* = 260)	d Period-1	Mean Period-2 (*n* = 1508)	d Period-2
Creativity	2.7545 (0.7558)	2.7262 (0.7593)	−0.0374	2.7325 (0.7294)	−0.0296
Curiosity	3.6560 (0.8352)	3.6071 (0.8704)	−0.0573	3.6562 (0.8498)	0.0002
Openness	3.5805 (0.7363)	3.4762 (0.8034)	−0.1354	3.6258 (0.7266) *	0.0619
Love of learning	3.8766 (0.7711)	3.8333 (0.7172)	−0.0581	3.9655 (0.7644) ***	0.1158
Perspective	3.5693 (0.7868)	3.5833 (0.8825)	0.0167	3.6035 (0.7978)	0.0432
Bravery	3.4188 (0.7585)	3.2857 (0.5571)	−0.2000	3.5005 (0.7674) ***	0.1071
Persistence	3.5829 (0.7935)	3.7024 (0.7105)	0.1587	3.6503 (0.7803) **	0.0856
Honesty	3.5257 (0.7452)	3.5833 (0.7941)	0.0748	3.6470 (0.7245) ***	0.1651
Zest	3.6204 (0.8731)	3.6905 (0.7477)	0.0862	3.7019 (0.8690) ***	0.0936
Intimacy	3.9707 (0.8146)	3.9643 (0.8081)	−0.0079	3.9161 (0.7936) *	−0.0679
Kindness	4.2555 (0.6953)	4.3571 (0.4963)	0.1682	4.3813 (0.6487) ***	0.1871
Social intelligence	3.5233 (0.7956)	3.0714 (0.7664) **	−0.5785	3.4648 (0.7716) **	−0.0746
**Teamwork**	**3.902 (0.6522)**	**4.1786 (0.6251) ***	**0.4330**	**4.0104 (0.6490) *****	**0.1666**
Fairness	3.6648 (0.8224)	3.7500 (0.7122)	0.1108	3.7286 (0.8188) **	0.0777
Leadership	3.4416 (0.8254)	3.369 (0.7822)	−0.0903	3.5030 (0.7960) **	0.0757
Forgiveness	3.3266 (0.9167)	3.1786 (0.8818)	−0.1646	3.3941 (0.9290) **	0.0731
Modesty	3.8067 (0.8069)	3.7500 (0.9585)	−0.0640	3.8207 (0.8256)	0.0172
Prudence	3.5994 (0.7867)	3.6548 (0.7453)	0.0723	3.6029 (0.7837)	0.0045
Self-control	3.4147 (0.8063)	3.4167 (0.7731)	0.0025	3.4749 (0.7813) **	0.0758
Appreciation of beauty	3.9402 (0.7783)	3.8095 (0.8034)	−0.1652	3.9902 (0.7325) *	0.0662
Gratitude	4.0452 (0.7323)	4.2976 (0.5690)	0.3849	4.0722 (0.7342)	0.0368
Hope	4.1694 (0.7856)	4.3690 (0.6109)	0.2836	4.2677 (0.7620) ***	0.1270
Humor	3.4417 (1.0092)	3.3810 (0.9968)	−0.0605	3.2831 (1.0075) ***	−0.1573
Spirituality	3.9428 (0.7075)	3.9762 (0.7366)	0.0462	3.9715 (0.7078)	0.0406
**(b) Gender-Controlled Set 2**					
**Traits**	**Mean** **Pre-Disaster** **(*n* = 5270)**	**Mean** **Period-1** **(*n* = 260)**	**d** **Period-1**	**Mean** **Period-2** **(*n* = 1508)**	**d** **Period-2**
Creativity	2.7400 (0.7556)	2.7262 (0.7593)	−0.0182	2.7403 (0.7642)	0.0004
Curiosity	3.6500 (0.8321)	3.6071 (0.8704)	−0.0504	3.6855 (0.8408)	0.0424
Openness	3.5630 (0.7380)	3.4762 (0.8034)	−0.1125	3.6371 (0.7395) ***	0.1003
Love of learning	3.8621 (0.7696)	3.8333 (0.7172)	−0.0387	3.9938 (0.7542) ***	0.1728
Perspective	3.5572 (0.7862)	3.5833 (0.8825)	0.0312	3.5976 (0.7963)	0.0511
Bravery	3.3963 (0.7645)	3.2857 (0.5571)	−0.1653	3.5055 (0.7921) ***	0.1403
Persistence	3.5627 (0.7900)	3.7024 (0.7105)	0.1859	3.6648 (0.79) ***	0.1292
Honesty	3.5223 (0.7444)	3.5833 (0.7941)	0.0793	3.6468 (0.7283) ***	0.1691
Zest	3.5994 (0.8745)	3.6905 (0.7477)	0.1120	3.7292 (0.8682) ***	0.1490
Intimacy	3.9663 (0.8088)	3.9643 (0.8081)	−0.0025	3.8905 (0.8044) ***	−0.0940
Kindness	4.2410 (0.7020)	4.3571 (0.4963)	0.1910	4.3907 (0.6393) ***	0.2230
Social intelligence	3.5108 (0.7960)	3.0714 (0.7664) **	−0.5624	3.4736 (0.7853)	−0.0470
**Teamwork**	**3.8965 (0.6554)**	**4.1786 (0.6251) ***	**0.4405**	**4.0185 (0.6345) *****	**0.1891**
Fairness	3.6557 (0.8259)	3.7500 (0.7122)	0.1223	3.7253 (0.8182) **	0.0847
Leadership	3.4269 (0.8276)	3.3690 (0.7822)	−0.0719	3.4918 (0.8243) **	0.0786
Modesty	3.7960 (0.8220)	3.7500 (0.9585)	−0.0515	3.8124 (0.84)	0.0197
Prudence	3.5808 (0.7950)	3.6548 (0.7453)	0.0960	3.6046 (0.8037)	0.0298
Self-control	3.3940 (0.8031)	3.4167 (0.7731)	0.0288	3.4828 (0.7798) ***	0.1122
Appreciation of beauty	3.9398 (0.7809)	3.8095 (0.8034)	−0.1645	3.9805 (0.7419)	0.0534
Gratitude	4.0357 (0.7322)	4.2976 (0.5690)	0.3994	4.0732 (0.7228)	0.0515
Hope	4.1629 (0.7913)	4.3690 (0.6109)	0.2916	4.295 (0.7446) ***	0.1719
Humor	3.434 (1.0094)	3.3810 (0.9968)	−0.0528	3.2546 (1.0307) ***	−0.1759
Spirituality	3.9333 (0.7054)	3.9762 (0.7366)	0.0595	3.9986 (0.7065) **	0.0925
**(c) Gender-Controlled Set 3**					
**Traits**	**Mean** **Pre-Disaster** **(*n* = 5270)**	**Mean** **Period-1** **(*n* = 260)**	**d** **Period-1**	**Mean** **Period-2** **(*n* = 1508)**	**d** **Period-2**
Creativity	2.7471 (0.7628)	2.7262 (0.7593)	−0.0275	2.7537 (0.7579)	0.0087
Curiosity	3.6431 (0.8382)	3.6071 (0.8704)	−0.0421	3.7012 (0.8471) *	0.0689
Openness	3.5727 (0.7399)	3.4762 (0.8034)	−0.1250	3.6433 (0.7457) ***	0.0950
Love of learning	3.8658 (0.7762)	3.8333 (0.7172)	−0.0435	4.0035 (0.7658) ***	0.1786
Perspective	3.5677 (0.7915)	3.5833 (0.8825)	0.0186	3.604 (0.8065)	0.0454
Bravery	3.4149 (0.7633)	3.2857 (0.5571)	−0.1934	3.498 (0.7918) ***	0.1069
Persistence	3.5652 (0.7980)	3.7024 (0.7105)	0.1816	3.6726 (0.8049) ***	0.1340
Honesty	3.5110 (0.7459)	3.5833 (0.7941)	0.0939	3.6509 (0.7425) ***	0.1880
Zest	3.5999 (0.8838)	3.6905 (0.7477)	0.1107	3.7328 (0.8746) ***	0.1512
Intimacy	3.9637 (0.8227)	3.9643 (0.8081)	0.0007	3.9452 (0.7991)	−0.0228
Kindness	4.2374 (0.7034)	4.3571 (0.4963)	0.1966	4.3991 (0.6515) ***	0.2385
Social Intelligence	3.5246 (0.7981)	3.0714 (0.7664) **	−0.5792	3.4703 (0.7964) *	−0.0681
**Teamwork**	**3.8935 (0.6571)**	**4.1786 (0.6251) ***	**0.4446**	**4.0144 (0.6477) *****	**0.1853**
Fairness	3.6627 (0.8219)	3.7500 (0.7122)	0.1135	3.7418 (0.8234) ***	0.0962
Leadership	3.4360 (0.8339)	3.3690 (0.7822)	−0.0829	3.5075 (0.8117) **	0.0869
Forgiveness	3.3138 (0.9057)	3.1786 (0.8818)	−0.1513	3.4011 (0.9316) ***	0.0950
Modesty	3.7872 (0.8183)	3.7500 (0.9585)	−0.0417	3.8279 (0.8335)	0.0493
Prudence	3.5925 (0.7934)	3.6548 (0.7453)	0.0809	3.6061 (0.8008)	0.0171
Self-control	3.3961 (0.7959)	3.4167 (0.7731)	0.0263	3.5114 (0.7931) ***	0.1451
Appreciation of beauty	3.9376 (0.7716)	3.8095 (0.8034)	−0.1626	4.009 (0.7325) ***	0.0949
Gratitude	4.0217 (0.7365)	4.2976 (0.5690) *	0.4192	4.076 (0.7394) **	0.0736
Hope	4.1467 (0.8010)	4.3690 (0.6109)	0.3121	4.2777 (0.7579) ***	0.1680
Humor	3.4320 (1.0134)	3.3810 (0.9968)	−0.0507	3.2826 (1.0296) ***	−0.1463
Spirituality	3.9305 (0.7134)	3.9762 (0.7366)	0.0630	3.9858 (0.7152) **	0.0774
**(d) Gender-Controlled Set 4**					
**Traits**	**Mean** **Pre-Disaster** **(*n* = 5270)**	**Mean** **Period-1** **(*n* = 260)**	**d** **Period-1**	**Mean** **Period-2** **(*n* = 1508)**	**d** **Period-2**
Creativity	2.7535 (0.7511)	2.7262 (0.7593)	−0.0361	2.7283 (0.7446)	−0.0337
Curiosity	3.6557 (0.8298)	3.6071 (0.8704)	−0.0572	3.6626 (0.847)	0.0082
Openness	3.5799 (0.7380)	3.4762 (0.8034)	0.1344	3.6449 (0.7306) **	0.0885
Love of learning	3.8705 (0.7764)	3.8333 (0.7172)	−0.0498	3.9727 (0.767) ***	0.1324
Perspective	3.5667 (0.793)	3.5833 (0.8825)	0.0198	3.5915 (0.8019)	0.0311
Bravery	3.4065 (0.7617)	3.2857 (0.5571)	−0.1810	3.488 (0.7731) ***	0.1062
Persistence	3.5793 (0.7909)	3.7024 (0.7105)	0.1637	3.6564 (0.7872) ***	0.0977
Honesty	3.5194 (0.7472)	3.5833 (0.7941)	0.0829	3.6406 (0.7335) ***	0.1637
Zest	3.6125 (0.8827)	3.6905 (0.7477)	0.0954	3.7194 (0.8691) ***	0.1220
Intimacy	3.9732 (0.8177)	3.9643 (0.8081)	−0.0109	3.9269 (0.8067) *	0.0570
Kindness	4.2512 (0.7032)	4.3571 (0.4963)	0.1740	4.3859 (0.6461) ***	0.1995
Social intelligence	3.5391 (0.7923)	3.0714 (0.7664)**	−0.6000	3.4711 (0.776) **	−0.0867
**Teamwork**	**3.8996 (0.6566)**	**4.1786 (0.6251) ***	**0.4352**	**4.0288 (0.6333) *****	**0.2003**
Fairness	3.6725 (0.8246)	3.7500 (0.7122)	0.1006	3.7314 (0.8144) *	0.0719
Leadership	3.4357 (0.8273)	3.3690 (0.7822)	−0.0829	3.5011 (0.8167) **	0.0796
Forgiveness	3.3151 (0.9110)	3.1786 (0.8818)	−0.1523	3.3933 (0.9284) **	0.0850
Modesty	3.8080 (0.8126)	3.7500 (0.9585)	−0.0653	3.8371 (0.8158)	0.0357
Prudence	3.5950 (0.7949)	3.6548 (0.7453)	0.0776	3.6213 (0.79)	0.0332
Self-control	3.4074 (0.8056)	3.4167 (0.7731)	0.0118	3.5089 (0.7769) ***	0.1283
Appreciation of beauty	3.9519 (0.7706)	3.8095 (0.8034)	−0.1809	4.0051 (0.7309) *	0.0708
Gratitude	4.0415 (0.7337)	4.2976 (0.5690)	0.3901	4.0817 (0.7321)	0.0549
Hope	4.1657 (0.7981)	4.3690 (0.6109)	0.2861	4.2867 (0.7585) ***	0.1554
Humor	3.4479 (1.0093)	3.3810 (0.9968)	0.0667	3.2664 (1.0188) ***	−0.1790
Spirituality	3.9390 (0.7095)	3.9762 (0.7366)	0.0514	4.0066 (0.7023) ***	0.0958
**(e) Gender-Controlled Set 5**					
**Traits**	**Mean** **Pre-Disaster** **(*n* = 5270)**	**Mean** **Period-1** **(*n* = 260)**	**d** **Period-1**	**Mean** **Period-2** **(*n* = 1508)**	**d** **Period-2**
Creativity	2.7436 (0.7583)	2.7262 (0.7593)	−0.0229	2.7638 (0.7511)	0.0268
Curiosity	3.6435 (0.8381)	3.6071 (0.8704)	−0.0426	3.686 (0.8408)	0.0506
Openness	3.5707 (0.7415)	3.4762 (0.8034)	−0.1222	3.6406 (0.7235) ***	0.0954
Love of learning	3.8622 (0.7761)	3.8333 (0.7172)	−0.0387	3.9739 (0.7661) ***	0.1449
Perspective	3.5591 (0.7924)	3.5833 (0.8825)	0.0289	3.6075 (0.8017) *	0.0607
Bravery	3.4056 (0.7605)	3.2857 (0.5571)	−0.1799	3.4997 (0.7841) ***	0.1218
Persistence	3.5667 (0.7900)	3.7024 (0.7105)	0.1806	3.6482 (0.7862) ***	0.1034
Honesty	3.5154 (0.7482)	3.5833 (0.7941)	0.0880	3.67 (0.7341) ***	0.2086
Zest	3.5953 (0.8817)	3.6905 (0.7477)	0.1165	3.7326 (0.8756) ***	0.1563
Intimacy	3.9640 (0.8205)	3.9643 (0.8081)	0.0004	3.892 (0.8023) **	−0.0887
Kindness	4.2382 (0.7045)	4.3571 (0.4963)	0.1951	4.3834 (0.6439) ***	0.2151
Social intelligence	3.5173 (0.8021)	3.0714 (0.7664) **	−0.5684	3.4824 (0.7800)	−0.0441
**Teamwork**	**3.8957 (0.6599)**	**4.1786 (0.6251) ***	**0.4401**	**3.9977 (0.6434) *****	**0.1565**
Fairness	3.6554 (0.8250)	3.7500 (0.7122)	0.1228	3.7182 (0.8267) **	0.0760
Leadership	3.4315 (0.8426)	3.3690 (0.7822)	−0.0769	3.5089 (0.8010) **	0.0942
Forgiveness	3.3014 (0.9053)	3.1786 (0.8818)	−0.1374	3.3783 (0.9337) **	0.0836
Modesty	3.7941 (0.8196)	3.7500 (0.9585)	−0.0495	3.8248 (0.8199)	0.0375
Prudence	3.5856 (0.7958)	3.6548 (0.7453)	0.0898	3.6168 (0.7897)	0.0394
Self-control	3.3940 (0.8066)	3.4167 (0.7731)	0.0287	3.5005 (0.7776) ***	0.1344
Appreciation of beauty	3.9333 (0.7838)	3.8095 (0.8034)	0.1560	4.0047 (0.7407) ***	0.0936
Gratitude	4.0194 (0.7401)	4.2976 (0.5690) *	0.4214	4.078 (0.7244) **	0.0800
Hope	4.1443 (0.8022)	4.3690 (0.6109)	0.3151	4.2775 (0.7512) ***	0.1714
Humor	3.4194 (1.0158)	3.3810 (0.9968)	−0.0382	3.2676 (1.0114) ***	−0.1498
Spirituality	3.9215 (0.7148)	3.9762 (0.7366)	0.0754	3.9819 (0.7021) **	0.0853

*Note*. Standard deviations are in parentheses. * means *p* < 0.05, ** means *p* < 0.01, *** means *p* < 0.001.

## References

[1] Murray D.R., Schaller M. (2010). Historical prevalence of infectious diseases within 230 geopolitical regions: A tool for investigating origins of culture. J. Cross-Cult. Psychol..

[2] Olivas-Lujan M.R., Harzing A.W., McCoy S. (2004). September 11, 2001: Two Quasi-experiments on the Influence of Threats on Cultural Values and Cosmopolitanism. Int. J. Cross Cult. Manag..

[3] Baino C., Zappullo I., Group T.L., Conson M. (2020). Tendency to worry and fear of mental health during Italy’s COVID-19 lockdown. Int. J. Environ. Res. Public Health.

[4] Zhou X. (2020). Psychological crisis interventions in Sichuan Province during the 2019 novel coronavirus outbreak. Psychiatry Res..

[5] Brooks S.K., Webster R.K., Smith L.E., Woodland L., Wessely S., Greenberg N., Rubin G.J. (2020). The psychological impact of quarantine and how to reduce it: Rapid review of the evidence. Lancet.

[6] Xiao C. (2020). A novel approach of consultation on 2019 novel coronavirus (COVID-19)-related psychological and mental problems: Structured letter therapy. Psychiatry Investig..

[7] Wang C., Pan R., Wan X., Tan Y., Xu L., Ho C.S., Ho R.C. (2020). Immediate psychological responses and associated factors during the initial stage of the 2019 Coronavirus Disease (COVID-19) epidemic among the general population in China. Int. J. Environ. Res. Public Health.

[8] Bonanno G.A. (2004). Loss, trauma, and human resilience: Have we underestimated the human capacity to thrive after extremely aversive events?. Am. Psychol..

[9] Guo J., Wang Y., Liu X.Y. (2015). Relation between marital satisfaction and character strengths in young people. Chin. Ment. Health J..

[10] Seligman M.E.P. (2002). Authentic Happiness: Using the New Positive Psychology to Realize Your Potential for Lasting Fulfillment.

[11] Peterson C., Seligman M.E.P. (2004). Character Strengths and Virtues: A Handbook and Classification.

[12] Gillham J., Adams-Deutsch Z., Werner J., Reivich K., Coulter-Heindl V., Linkins M., Contero A. (2011). Character strengths predict subjective well-being during adolescence. J. Posit. Psychol..

[13] Park N., Peterson C., Seligman M.E. (2004). Strengths of character and well-being. J. Soc. Clin. Psychol..

[14] Proctor C., Maltby J., Linley P.A. (2011). Strengths Use as a Predictor of Well-Being and Health-Related Quality of Life. J. Happiness Stud..

[15] Wang Y., Liang C.G. (2017). Relationship among character strength, life stress events and emotional well-being in nursing staffs. Chin. Occup. Med..

[16] Petkari E., Ortiz-Tallo M. (2018). Towards youth happiness and mental health in the United Arab Emirates: The path of character strengths in a multicultural population. J. Happiness Stud..

[17] Freidlin P., Littman-Ovadia H., Niemiec R.M. (2017). Positive psychopathology: Social anxiety via character strengths underuse and overuse. Personal. Individ. Differ..

[18] Huta V., Hawley L. (2010). Psychological strengths and cognitive vulnerabilities: Are they two ends of the same continuum or do they have independent relationships with well-being and ill-being?. J. Happiness Stud..

[19] Kim H.R., Kim S.M., Hong J.S., Han D.H., Yoo S.K., Min K.J., Lee Y.S. (2018). Character strengths as protective factors against depression and suicidality among male and female employees. BMC Public Health.

[20] Tehranchi A., Neshat Doost H.T., Amiri S., Power M.J. (2018). The role of character strengths in depression: A structural equation model. Front. Psychol..

[21] Chen R., Sun C., Chen J., Jen H., Kang X., Kao C., Chou K. (2021). A large-scale survey on trauma, burnout, and posttraumatic growth among nurses during the COVID-19 pandemic. Int. J. Ment. Health Nurs..

[22] Gander F., Wagner L. (2022). Character growth following collective life events: A study on perceived and measured changes in character strengths during the first wave of the COVID-19 pandemic. Eur. J. Personal..

[23] Rashid T., McGrath R.E. (2020). Strengths-based actions to enhance wellbeing in the time of COVID-19. Int. J. Wellbeing.

[24] Stallard P., Pereira A., Barros L. (2021). Post-traumatic growth during the COVID-19 pandemic in carers of children in Portugal and the UK: Cross-sectional online survey. BJPsych Open.

[25] Yu Y., Yu Y., Hu J. (2022). COVID-19 among Chinese high school graduates: Psychological distress, growth, meaning in life and resilience. J. Health Psychol..

[26] Liu Q., Wang Z. (2021). Perceived stress of the COVID-19 pandemic and adolescents’ depression symptoms: The moderating role of character strengths. Personal. Individ. Differ..

[27] Umucu E., Tansey T.N., Brooks J., Lee B. (2021). The protective role of character strengths in COVID-19 stress and well-being in individuals with chronic conditions and disabilities: An exploratory study. Rehabil. Couns. Bull..

[28] Casali N., Feraco T., Meneghetti C. (2021). Character strengths sustain mental health and post-traumatic growth during the COVID-19 pandemic. A longitudinal analysis. Psychol. Heath.

[29] Vasileiou D., Moraitou D., Papaliagkas V., Pezirkianidis C., Stalikas A., Papantoniou G., Sofologi M. (2021). The relationships between character strengths and subjective wellbeing: Evidence from Greece under lockdown during COVID-19 pandemic. Int. J. Environ. Res. Public Health.

[30] Martinez-Marti M.L., Theirs C.I., Pascual D., Corradi G. (2020). Character strengths predict an increase in mental health and subjective well-being over a one-month period during the COVID-19 pandemic lockdown. Front. Psychol..

[31] Peterson C., Seligman M.E.P. (2003). Character strengths before and after September 11. Psychol. Sci..

[32] Peterson C., Park N., Seligman M.E.P. (2006). Greater strengths of character and recovery from illness. J. Posit. Psychol..

[33] Peterson C., Park N., Pole N., D’Andrea W., Seligman M.E.P. (2008). Strengths of character and posttraumatic growth. J. Trauma. Stress.

[34] Schueller S.M., Jayawickreme E., Blackie L.E.R., Forgeard M.J.C., Roepke A.M. (2015). Finding character strengths through loss: An extension of Peterson and Seligman (2003). J. Posit. Psychol..

[35] Niemiec R.M. (2019). Six functions of character strengths for thriving at times of adversity and opportunity: A theoretical perspective. Appl. Res. Qual. Life.

[36] Triandis H.C. (1996). The psychological measurement of cultural syndromes. Am. Psychol..

[37] Triandis H.C. (1989). The self and social behavior in different cultural contexts. Psychol. Rev..

[38] Casali N., Feraco T., Ghisi M., Meneghetti C. (2020). “Andrà tutto bene”: Associations between character strengths, psychological distress and self-efficacy during COVID-19 lockdown. J. Happiness Stud..

[39] Park N., Peterson C., Ong A.D., van Dulmen M. (2006). Methodological issues in positive psychology and the assessment of character strengths. Handbook of Methods in Positive Psychology.

[40] Duan W., Ho S.M., Yu B., Tang X., Zhang Y., Li T., Yuen T. (2012). Factor structure of the Chinese virtues questionnaire. Res. Soc. Work Pract..

[41] Wu J.J. (2014). Development of the Positive Mental Characters Scale for College Students. China J. Health Psychol..

[42] Zang Y.H., Yang J., Wu L. (2017). The structure validation of positive psychology character scale about the poor college students. Psychol. Explor..

[43] Meng W.J., Guan Q. (2009). Development of the positive mental characters scale for Chinese college students. Chin. J. Spec. Educ..

[44] Guan Q., Meng W.J., Keller J. (2009). Development of the positive mental characters scale for Chinese school students. Chin. J. Spec. Educ..

[45] Murray D.R., Schaller M. (2016). The behavioral immune system: Implications for social cognition, social interaction, and social influence. Adv. Exp. Soc. Psychol..

[46] Schaller M. (2011). The behavioral immune system and the psychology of human sociality. Philos. Trans. R. Soc. B Biol. Sci..

[47] Schaller M., Park J.H. (2011). The behavioral immune system (and why it matters). Curr. Dir. Psychol. Sci..

[48] Faulkner G., Schaller M., Park J.H., Duncan L.A. (2004). Evolved disease-avoidance mechanisms and contemporary xenophobic attitudes. Group Process. Intergroup Relat..

[49] Gaertner S.L., Dovidio J.F. (2000). Reducing Intergroup Bias: The Common Ingroup Identity Model.

[50] Murray D.R., Schaller M. (2012). Threat(s) and conformity deconstructed: Perceived threat of infectious disease and its implications for conformist attitudes and behavior. Eur. J. Soc. Psychol..

[51] Fincher C.L., Thornhill R., Murray D.R., Schaller M. (2008). Pathogen prevalence predicts human cross-cultural variability in individualism/collectivism. Proc. R. Soc. B Biol. Sci..

[52] Grossmann I., Varnum M.E.W. (2015). Social structure, infectious diseases, disasters, secularism and cultural change in America. Psychol. Sci..

[53] Oishi S., Komiya A. (2017). Natural disaster risk and collectivism. J. Cross-Cult. Psychol..

[54] Zhao L., Ding X., Yu F. (2020). Public moral motivation during the COVID-19 pandemic: Analysis of posts on Chinese social media. Soc. Behav. Personal..

[55] Haidt J. (2012). The Righteous Mind: Why Good People Are Divided by Politics and Religion.

[56] Park N., Peterson C. (2006). Moral competence and character strengths among adolescents: The development and validation of the Values in Action Inventory of Strengths for Youth. J. Adolesc..

[57] Taylor S.E., Klein L.C., Lewis B.P., Gruenewald T.L., Gurung R.A.R., Updegraff J.A. (2000). Biobehavioral responses to stress in females: Tend-and-befriend, not fight-or-flight. Psychol. Rev..

[58] Hu L., Bentler P. (1999). Cut-off criteria for fit indexes in covariance structure analysis: Conventional criteria versus new alternatives. Struct. Equ. Model..

[59] Pezirkianidis C., Karakasidou E., Stalikas A., Moraitou D., Charalambous V. (2020). Character strengths and virtues in the Greek cultural context. Psychol. J. Hell. Psychol. Soc..

[60] Agrawal R., Imielinski T., Swami A. Mining Association Rules between Sets of Items in Large Databases. Proceedings of the ACM SIGMOD International Conference on Management of Data.

[61] Naushad V.A., Bierens J.J.L.M., Nishan K.P., Firjeeth C.P., Mohammad O.H., Maliyakkal A.M., Chalihadan S., Schreiber M.D. (2019). A systematic review of the impact of disaster on the mental health of medical responders. Prehospital Disaster Med..

[62] Kowal M., Karwowski M., Coll-Martin T., Ikizer G., Rasmussen J., Lieberoth A., Eichel K., Studziska A., Koszakowska K., Karwowski M. (2020). Who is the most stressed during the COVID-19 pandemic? Data from 26 countries and areas. Appl. Psychol. Health Well-Being.

[63] Brent Donnellan M., Robins R., Corr P., Matthews G. (2009). The development of personality across the lifespan. The Cambridge Handbook of Personality Psychology.

[64] Kritzler S., Rakhshani A., Terwiel S., Fassbender I., Donnellan B., Lucas R.E., Luhmann M. (2021). How are common major live events perceived? Exploring differences between and variability of different typical event profiles and raters. PsyArXiv.

